# Exploring Global Strategies to Combat Rabies: Addressing Limitations and Enhancing Effectiveness

**DOI:** 10.1002/vms3.70669

**Published:** 2025-10-18

**Authors:** Jennifer F. Lyabangi, Elton M. Mahulu, Jossiah Sandetwa, Withness A. John, Philbert B. Madoshi

**Affiliations:** ^1^ St. Francis University College of Health and Allied Sciences (SFUCHAS) Morogoro Tanzania

**Keywords:** control strategies, public health, rabies, vaccination, zoonotic diseases

## Abstract

Rabies is a fatal viral disease affecting the central nervous system of warm‐blooded animals including humans; rabies remains a significant global health threat, with over 99% of human cases transmitted through, resulting in approximately 55,000 deaths annually, mainly in Africa and Asia. Rabies manifests in two forms: one characterized by agitation and hydrophobia, and the other, paralytic, marked by muscle weakness and paralysis. The article stresses on the effectiveness of control strategies, including the One Health approach, mass dog vaccination and oral vaccination in line with the ‘Zero by 30’ initiative, which seeks to eliminate dog‐mediated rabies deaths by 2030. However, enforcement, access to post‐exposure prophylaxis (PEP), poor dog population management and weak surveillance continue to impede progress, particularly in low‐ and middle‐income countries. Strengthening collaboration across policymakers, health professionals, public health sectors and environmental health sectors is crucial to reducing the rabies burden and achieving global health objectives. This article aims to explore the global strategies and challenges in controlling and preventing rabies.

## Graphical abstract

Rabies causes 55,000 deaths annually, mainly in Africa and Asia, despite being preventable. Global strategies such as the One Health approach, mass dog vaccination and PEP aim to eliminate dog‐mediated human rabies by 2030. Gaps in enforcement, dog population control and surveillance hinder progress, especially in low‐resource settings.

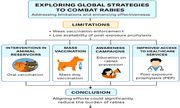

## 1 Introduction

Rabies is an infectious viral disease associated with inflammation of the central nervous system; it affects all mammals including humans (PAHO/WHO 2024). It is one of the potentially underrated zoonotic diseases causing global health threat. More than 99% of all human rabies cases are transmitted by dog bites, whereby every year 55,000 humans and countless dogs die from rabies, mostly in Africa and Asia (PAHO/WHO 2024). Besides rabies being preventable before and after exposure, the strategies have not been effective in most of the developing countries. In addition, even in countries that have claimed to eliminate the disease, its success can be fragile since the interventions are regularly either weakened or stopped (Fahrion et al. 2023).

Rabies presents in two forms: encephalitic and paralytic rabies. Encephalitic rabies presents with agitation, confusion and hydrophobia (PAHO/WHO 2024). Paralytic rabies presents with muscle weakness, paralysis and coma. The most complications caused by rabies are due to involvement of the central nervous system after infection with the virus and eventually lead to death in nearly 100% of patients (Jin 2023).

## 2 Interventions and Preventions

Rabies is a significant public health concern, particularly in low‐ and middle‐income countries, where the burden of the disease is disproportionately high. The One Health approach, which integrates human, animal and environmental health, has been recognized as a pragmatic and equitable strategy to address global health challenges (Shafaati et al. 2023). The strategy has been proposed to be cost‐effective in rabies control, aiming to reduce the high number of annual rabies deaths. This approach aligns the needs of disadvantaged communities with global health goals, contributing to sustainable development and global health security (Cleaveland et al. 2017). The practical application of the One Health approach has been emphasized by various organizations, leading to the establishment of expert panels to address zoonotic diseases like rabies (Tidman 2022). Successful interventions, such as the elimination of rabies virus variants in wildlife through oral vaccination (oral baiting), demonstrate the potential for controlling rabies in animal reservoirs (Slate et al. 2009).

Furthermore, the implementation of national rabies control strategies, aligned with the ‘Zero by 30’ global goal to end dog‐mediated human rabies deaths by 2030, exemplifies the commitment to combat rabies at a global level (Athingo et al. 2020). Vaccination plays a crucial role in rabies control, with studies highlighting the induction of protective antibodies following oral immunization in wildlife (Moore et al. 2017). In addition, scoping reviews emphasize the effectiveness of control interventions, including mass dog vaccination, to achieve the goal of zero canine‐mediated human rabies deaths by 2030 (Safii and Abdul Taib 2020). Understanding the contact network structures of dog populations is essential for designing effective mass vaccination strategies to prevent rabies transmission (Laager et al. 2018). Achieving herd immunity is central to rabies elimination. Evidence consistently shows that vaccinating at least 70% of the dog population interrupts rabies transmission and provides community‐wide protection (Lugelo et al. 2022). This threshold is globally recognized as the cornerstone of canine rabies control (Coleman and Dye 1996). In general, awareness and education are vital components of rabies prevention, as evidenced by studies focusing on rural communities and school students to improve protective behaviours and healthcare utilization following animal bites (Sikana et al. 2021). However, Community risk perception greatly influences rabies prevention and control. Misconceptions about dog bites, cultural practices surrounding traditional healers and low awareness of PEP often delay treatment‐seeking behaviours (Rana et al. 2021). Enhancing community knowledge and perception of rabies risk is essential to improving compliance with PEP and vaccination (Santhaseelan et al. 2024). Geographic access to care and the provisioning of PEP are critical factors in shaping the disease burden and preventing human rabies deaths (Rajeev et al. 2021).

### 2.1 Surveillance Systems

Effective rabies control requires robust surveillance systems to monitor the disease and guide targeted interventions (WHO–Eastern Mediterranean Regional n.d.). Surveillance enables identification of high‐risk areas, helps in quantification of the disease burden and so informing on intervention to be employed (Shah and Househ 2024). Passive surveillance, which relies on reports from healthcare or veterinary facilities, helps in detecting trends but often misses some information due to underreporting (Murray and Cohen 2016). In contrast, active surveillance involves field investigation, sample collection from suspected rabid animals and laboratory confirmation, hence providing more accurate epidemiological data (Murray and Cohen 2016). Integration of both forms is essential in ensuring timely detection, resource allocation and evaluation of interventions.

### 2.2 Limitations of Current Strategies

Despite the current efforts made to mitigate the burden of rabies in the world, there remain gaps in making the efforts effective. In most countries, the effectiveness of vaccination is weak due to failure to enforce vaccination consistently and poor implementation of policies, leaving many individuals susceptible to contracting rabies (Haselbeck et al. 2021). Also, due to insufficient resources allocated in the fight against the disease, it becomes very challenging to implement comprehensive control strategies, especially in resource‐limited settings (Taylor and Partners for Rabies Prevention 2013).

Also, unavailability and unaffordability of PEP in most communities, especially in low‐ and middle‐income countries, have led to increasing mortality due to rabies. Also, due to a lack of clear guidelines for PEP administration, there is vaccination of people with no risk, leaving people with risk contacts without vaccination (Changalucha et al. 2019).

Furthermore, the failure to control the disease in free‐roaming dogs is another significant issue, which tends to increase the burden of the disease. Improper dog population management and weak surveillance have also contributed to the persistence of rabies (Tiwari et al. 2021). Also, the lack of good collaboration between human, animal and environmental health specialties can also impede the effectiveness of the applied control strategies, since the One Health approach has shown significant effectiveness in some countries (Haselbeck et al. 2021). Most of the current strategies rely on human and animal components, neglecting the role of environmental management in rabies prevention. Poor waste management contributes to sustaining free‐roaming dog population, while human encroachment on wildlife habitats increases the risk of spillover at human–animal interface (Aga et al. 2016). Strengthening environmental management alongside vaccination and surveillance is therefore essential within the One Health framework (Acharya et al. 2020).

### 2.3 Stakeholders’ Involvement

Multisectoral stakeholder involvement is necessary to ensure effective designing and sustaining effective control programs (Taylor and Partners for Rabies Prevention 2013). The One Health framework has demonstrated the effectiveness of the program, where the convergence of human, animal and environmental health priorities has increased program impact and resource efficiency (CDC 2025). Engaging stakeholders not only improves policies and public compliance but also ensures sustainability of the programs (Nel 2018).

## 3 Conclusion

In conclusion, the exploration of global strategies to combat rabies involves a multidisciplinary approach, integrating interventions in animal reservoirs, dog mass vaccination, awareness campaigns and improved access to healthcare services. These efforts could align with the ‘Zero by 30’ global campaign and demonstrate the potential to significantly reduce the burden of rabies, particularly in low‐ and middle‐income countries.

## Author Contributions

**Jennifer F. Lyabangi**: conceptualization, methodology, software, data curation. **Elton M. Mahulu**: investigation, validation, formal analysis, writing – review and editing. **Josiah I. Sandetwa**: investigation, validation, formal analysis, resources. **Witness A. John**: formal analysis, supervision, funding acquisition, project administration. **Philbert B. Madoshi**: writing – review and editing, supervision, formal analysis.

## Ethics Statement

The authors have nothing to report.

## Conflicts of Interest

The authors declare no conflicts of interest.

## Peer Review

The peer review history for this article is available at https://www.webofscience.com/api/gateway/wos/peer‐review/10.1002/vms3.70669.

## Data Availability

The authors have nothing to report.
